# CD4^+^CD25^+^FOXP3^+^ Regulatory T Cells Suppress Anti-Tumor Immune Responses in Patients with Colorectal Cancer

**DOI:** 10.1371/journal.pone.0000129

**Published:** 2006-12-27

**Authors:** Sarah L. Clarke, Gareth J. Betts, Andrea Plant, Kate L. Wright, Tariq M. El-Shanawany, Richard Harrop, Jared Torkington, Brian I. Rees, Geraint T. Williams, Awen M. Gallimore, Andrew J. Godkin

**Affiliations:** 1 Department of Medical Biochemistry and Immunology, Cardiff University, Cardiff, United Kingdom; 2 Oxford Biomedica, Oxford, United Kingdom; 3 Department of Surgery, University Hospital of Wales, Cardiff, United Kingdom; 4 Department of Pathology, Cardiff University, Cardiff, United Kingdom; Sanofi-Aventis, United States of America

## Abstract

**Background:**

A wealth of evidence obtained using mouse models indicates that CD4^+^CD25^+^FOXP3^+^ regulatory T cells (Treg) maintain peripheral tolerance to self-antigens and also inhibit anti-tumor immune responses. To date there is limited information about CD4^+^ T cell responses in patients with colorectal cancer (CRC). We set out to measure T cell responses to a tumor-associated antigen and examine whether Treg impinge on those anti-tumor immune responses in CRC patients.

**Methodology and Principal Findings:**

Treg were identified and characterized as CD4^+^CD25^+^FOXP3^+^ using flow cytometry. An increased frequency of Treg was demonstrated in both peripheral blood and mesenteric lymph nodes of patients with colorectal cancer (CRC) compared with either healthy controls or patients with inflammatory bowel disease (IBD). Depletion of Treg from peripheral blood mononuclear cells (PBMC) of CRC patients unmasked CD4^+^ T cell responses, as observed by IFNγ release, to the tumor associated antigen 5T4, whereas no effect was observed in a healthy age-matched control group.

**Conclusions/Significance:**

Collectively, these data demonstrate that Treg capable of inhibiting tumor associated antigen-specific immune responses are enriched in patients with CRC. These results support a rationale for manipulating Treg to enhance cancer immunotherapy.

## Introduction

Colorectal cancer (CRC) is the fourth most commonly diagnosed malignant disease with an estimated 1 million new cases and over 500 000 deaths each year worldwide [1]. The current management of patients with CRC revolves around excision of the primary tumor and the local lymph nodes and the selective use of 5-fluorouracil (5FU; inhibits thymidylate synthase) based chemotherapy [2]. Recent advances in preoperative imaging, surgical technique and the use of neo-adjuvant chemotherapy have improved outcomes, but colorectal cancer remains the second leading cause of death from cancer in Western countries with 40–50% of patients who undergo a potentially curative operation, undergoing relapse or death from metastatic disease.

Clinico-pathological staging of the disease strongly correlates with prognosis. Interestingly, improved clinical outcome is also associated with the presence of tumor-infiltrating lymphocytes (TILs), particularly if the lymphocytes invade the glandular elements of the tumor [3], [4]. This suggests that anti-tumor immune responses can impinge on the growth of the primary tumor, and may have an important role in controlling metastatic disease. Indirect evidence for the importance of the immune response in cancer patients is the epidemiological association in some populations between an increased incidence of tumors and immunosuppression post organ transplantation [5].

Much interest has recently arisen in the role of a naturally occurring population of CD4^+^CD25^+^ regulatory T cells (Treg), characterized by expression of the forked-head transcription factor FOXP3, and increased levels of the surface markers CD45RO, CTLA-4, and GITR [6], [7], [8]. Treg have been shown to control self-antigen specific responses in the periphery, and hence may play a role in controlling anti-tumor immune responses. Several groups including our own, have demonstrated that depletion of Treg promotes the generation of anti-tumor immune responses and tumor rejection in murine models (reviewed in [9]). There is evidence to suggest that these protective responses target both tumor-specific antigens as well as shared tumor antigens [10], [11].

More recently, several studies have observed an increased frequency of CD4^+^CD25^+^ T cells in the peripheral blood of patients with various types of cancer, although the majority of these studies did not confirm that these were Treg by staining for FOXP3 (reviewed in [9]). The presence of FOXP3^+^ Treg within the TILs of ovarian cancer has, however, been identified as an independent risk factor for poorer prognosis. Furthermore, after purification from tumor ascites, these Treg were shown to suppress Her2-specific T cell responses *in vitro* [12].

The human onco-fetal antigen, 5T4, is a 72kDa leucine-rich membrane glycoprotein which is expressed at high levels on the placenta and also on a wide range of human carcinomas including colorectal, gastric, renal and ovarian but rarely on normal tissues [13], [14], [15], [16], [17], [18]. In this study we tested the hypothesis that Treg develop in CRC patients that suppress 5T4-specific immune responses. Samples from patients with CRC or IBD, and healthy controls were examined to address three questions: is the frequency of Treg (CD25^hi^CD4^+^FOXP3^+^ T cells) increased in blood or lymph nodes from patients with CRC; can anti-tumor T cell responses to 5T4 be identified in CRC patients; and do Treg impinge on these anti-tumor immune responses?

## Methods

### Sample groups

CRC patients were identified from multi-disciplinary team meetings with the first presentation of an adenocarcinoma and no reported distant metastases on cross sectional imaging (TNM stage I–III; Duke's stage A–C). IBD patients (ulcerative colitis or Crohn's disease) attending clinic or undergoing elective surgical resection were recruited to the study. Local ethical committee approval was granted for the study by the Bro Taf Local Research Ethics Committee.

### Antigens

Purified protein derivative (PPD) (Statens Serum Institut, Denmark), hemaglutinin (HA) was a kind gift from Dr John Skehel (NIMR, Mill Hill), 5T4 was produced as described previously [19] (Oxford Biomedica). All antigens were used at a final concentration of 1 µg/ml.

### Purification of lymphocytes

30–50 ml of blood was drawn from patients or controls and heparinized (10 U/ml). PBMCs were extracted by centrifugation over Lymphoprep (Axis-Shield, Oslo, Norway). The cells were washed twice in RPMI before further use. Lymph nodes were dissected from fresh specimens within 30 minutes of surgery, washed in RPMI, mashed through a sterile cell strainer and collected by centrifugation.

### Flow cytometric analysis of lymphocytes from blood and lymph nodes

Samples were stained with fluorescently-labeled antibodies to CD4 (clone RPA-T4), CD25 (clone M-A251), CD45RO (clone UCHL1) and CTLA4 (clone BNI3) (BD Pharmingen), CD4 (clone S3.5) (Caltag) and CD25 (clone 4E3) (Miltenyi Biotec). All staining was performed in phosphate buffered saline (PBS), 2.5% FCS, 5 mM EDTA and intracellular staining was performed using the cytofix, cytoperm kit (BD Pharmingen). FOXP3 staining was performed using the FITC anti-human FOXP3 staining kit (clone PCH101) (71-5776 eBioscience). Lymphocytes were gated on forward and side scatter profiles. All flow cytometry was performed on a BD FacsCalibur and analyzed using the Summit v3.1 analysis program (build 839 Dakocytomation, Fort Collins, Colorado).

### CD4^+^CD25^hi^ add-back experiments

Purified PBMC were enriched for CD4^+^ cells and then separated into CD4^+^CD25^hi ^and CD4^+^CD25^−^ fractions using magnetic beads (Miltenyi CD4^+^CD25^+^ regulatory isolation kit). Flow cytometry of each fraction showed that the stringency of CD25^+^ cell selection by the Miltenyi CD4^+^CD25^+^ regulatory isolation kit and Miltenyi anti-CD25 beads was comparable. A proliferation assay was set up using 2x10^4^ CD4^+^CD25^−^ cells, activated with 0.5 µg/ml plate-bound anti-CD3 (clone OKT3) (eBioscience) and 1 µg/ml soluble anti-CD28 (clone 28.2) (BD Pharmingen) antibodies in RPMI 1640 supplemented with 10% heat-treated FCS, 100 U/ml penicillin, 100 µg/ml streptomycin, 2 mM L-glutamine and 1 mM sodium pyruvate. CD4^+^CD25^−^ cells were activated alone or in the presence of CD4^+^CD25^hi^ at cells varying ratios of 1∶1, 1∶0.1, 1∶0.01 and 1∶0.001 CD4^+^CD25^−^∶CD4^+^CD25^hi^ cells. Proliferation was measured on day 3 by the addition of 0.037 MBq/well (1 µCI) of [*methyl*-3H]-thymidine (Amersham Biosciences) for the final 18 hours of culture. Cells were harvested onto printed filtermat A filters (Wallac, Turku, Finland supplier Perkin Elmer) using a TomTek cell harvester. Once filters were dry, meltilex A melt-on scintillator sheets (Wallac) were added and filters were read using a Trilux 1450 microbeta liquid scintillation and luminescence counter (Wallac).

### IFN-γ ELISPOT assays

Antibodies were obtained from Mabtech (Natka, Sweden) and the ELISPOT was developed using the alkaline phosphatase substrate kit from Bio-Rad (Hercules, California). The effect of Treg depletion on antigen-specific IFN-γ production was assessed in parallel assays. CD25^hi^ cells were depleted using magnetic separation with MACs CD25 microbeads (Miltenyi Biotec, Germany). Briefly, 1.5×10^7^ PBMC were incubated with 15 µl of anti-CD25 coated beads in total reaction volume of 150 µl of buffer (1×PBS, 0.5% BSA, 5 mM EDTA) at 4°C for 15 min. Excess beads were washed off and the PBMC were run down an MS column. The column was washed with 3×1 ml washes in buffer. The efficacy of depletion was confirmed by flow cytometry. Assays were set up using either 3.5×10^5^ undepleted or CD25^hi^-depleted cells in RPMI 1640 supplemented with 5% heat-treated FCS, 100 U/ml penicillin, 100 µg/ml streptomycin, 2 mM L-glutamine and 1 mM sodium pyruvate per well of a polymer-backed 96-well filtration plate (MAIP-S-4510) (Millipore, Moslheim, France) in a total reaction volume of 100 µl/well. The concentrations of antibodies used and washing steps were according to the manufacturer's instructions, all antibody incubations were with 50 µl/well. The method has been previously described [20]. PPD, HA and 5T4 were tested in duplicate wells, having been added to a final concentration of 1 µg/ml, and compared with control wells with no protein. The plate was incubated at 37°C, 5% CO_2_ for 18 h. Activated T cells were enumerated at the single-cell level by counting the number of spots per well using an automated ELISPOT reader (Cadama). Responders were identified as having at least 5 spot-forming cells (sfc) per 10^6^ PBMC, after subtraction of the background, and an increase of at least 50% above background.

### Statistical analysis

Flow cytometric data was analyzed using the unpaired student's *t*-test (GraphPad Prism version 2.0 (GraphPad software). The proportion of cancer patients and healthy controls mounting a response to 5T4 was compared using a closed form method for comparing differences and calculating confidence intervals [21].

## Results

### Defining the human Treg population

**Figure 1:**
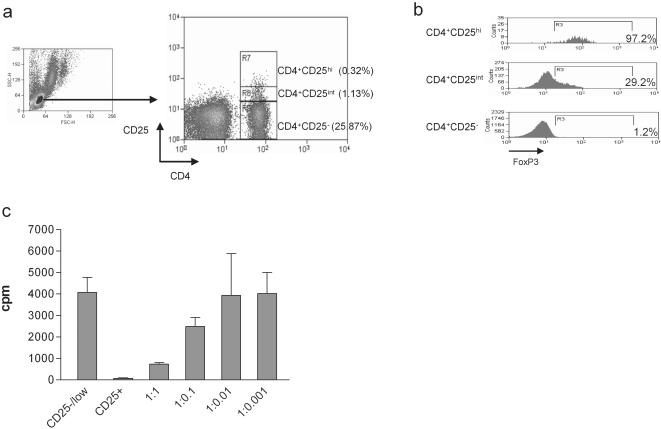
Characterization of the human Treg population. (A) CD4^+^CD25^−^, CD4^+^CD25^int^ and CD4^+^CD25^hi^ cells were identified as shown. CD4^+^CD25^hi^ cells are those where CD25 expression was higher than that on the CD4^−^ population. (B) FOXP3 expression within each population was assessed by intracellular staining. (C) Freshly isolated CD4^+^CD25^−^ T cells (2×10^4^ cells/well) were cultured alone (CD25-/low) or with CD4^+^CD25^hi^ T cells at various ratios, and stimulated with plate-bound anti-CD3 and soluble anti-CD28. Proliferation was assessed by (^3^H)-thymidine incorporation. The results represent the average of triplicate wells with standard deviation, from a representative assay.

Using the strict gating parameters reported recently [8], Treg were identified as CD4 positive cells with brighter CD25 staining than that of the CD4-negative population (Fig 1a). The frequency of Treg was calculated as the percentage of CD4^+^CD25^hi^ cells in the CD4^+^ population, and was in the range of 0.09–1.44% of CD4^+^ cells in PBMC of healthy age-matched controls. In keeping with previous reports, the vast majority of CD4^+^CD25^hi^ cells were CTLA4-positive and expressed CD45RO (>97%) [6], [7], [8]. The CD25^hi^ population was further analyzed by the expression of FOXP3. As shown in Figure 1b, over 95% of CD4^+^CD25^hi^ cells expressed FOXP3, compared with approximately 30% of CD4^+^CD25^int^ cells. This pattern of staining was the same for both healthy controls and CRC patients. FOXP3 expression was negligible in CD4^+^CD25^−^ cells (Figure 1b).

To confirm the regulatory phenotype of this population of CD4^+^CD25^hi^ cells, their ability to suppress the activation of CD4^+^CD25^−^ cells was assessed using a co-culture *in vitro* proliferation assay. CD4^+^CD25^−^ and CD4^+^CD25^hi^ cells were separated from PBMC, as described in methods. The addition of CD4^+^CD25^hi^ cells to polyclonally stimulated CD4^+^ T cells clearly suppressed the proliferation of CD4^+^CD25^−^ T cells and, as reported by others, this suppression was dose-dependent [8], [22], [23] (Figure 1c).

### CRC patients have increased numbers of Treg in peripheral blood

**Figure 2:**
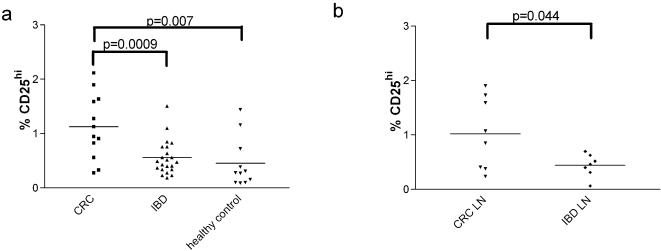
CRC patients have increased numbers of Treg cells. (A) Freshly isolated PBMC from 12 CRC patients, 11 healthy age-matched controls and 22 IBD patients were stained with antibodies to CD4 and CD25. Using the gating strategy outlined in Figure 1, Treg were enumerated. (B) Mesenteric lymph node samples (LN) were collected from surgical specimens of 8 CRC and 7 IBD patients and stained and gated as above. The data show the percentage of CD4^+^ cells that were CD25^hi^ in each sample. Each symbol represents one patient. The p values were calculated using an unpaired student's *t*-test.

The Treg were phenotypically defined as outlined above. Figure 2a shows the frequencies of Treg in the PBMCs of CRC patients (n = 12, age range 58–80 years) compared with age-matched healthy controls (n = 11, age range 48–93 years) and patients with inflammatory bowel disease (n = 22, age range 19–80 years). In all groups, the frequency of Treg was less than 2% of CD4^+^ T cells. However, the mean frequency in CRC patients (1.13%) was significantly increased compared with the control groups (IBD patients 0.56%, healthy controls 0.46%). There was no significant difference in the frequency of Treg in healthy controls and IBD patients.

### CRC patients have increased numbers of Treg in mesenteric lymph nodes

Obtaining control lymph nodes from subjects with no abdominal disease was not possible. IBD patients offer a colonic disease control group to the CRC patients, the latter also often demonstrating areas of inflammation around the tumor. Furthermore, the results from PBMCs did not demonstrate a significant alteration in the frequencies of Treg in patients with IBD compared with healthy controls. Hence, we considered it reasonable to use mesenteric lymph nodes obtained from IBD patients undergoing surgery as a control group in which to examine Treg in secondary lymphoid tissue.

Fresh lymph nodes were obtained from the surgically resected specimens and prepared as outlined in methods. The frequencies of Treg in mesenteric lymph nodes obtained from CRC patients (n = 8), and IBD patients (n = 7) were compared (Figure 2b). CRC patients again showed a significant increase in the frequency of Treg, reflecting the increase already described in peripheral blood. The lymph node-derived CD25^hi^ Treg had the same phenotype to those found in blood (data not shown). No difference was observed in the overall proportion of CD4^+^ or CD8^+^ T cells (data not shown).

### Patients with non-metastatic CRC demonstrate vigorous CD4^+^ T cell responses to recall antigens

**Figure 3:**
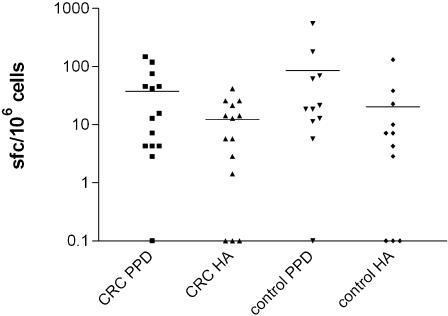
CRC patients are able to mount CD4^+^ T cell responses to the recall antigens PPD and HA. Whole PBMC from 11 age-matched controls and 14 CRC patients were assessed for responses to the antigens PPD and HA, measured by IFN-γ ELISPOT. Purified PBMC were added at 3.5×10^5^ cells/well, and incubated for 18 hours in the presence of either 1 µg/ml PPD, 1 µg/ml HA or with no antigen. Wells were set up in duplicate. Fewer than 5 spot forming cells/10^6^ PBMC was considered negative.

PBMCs were purified from CRC patients (n = 14) prior to surgery and the specific CD4^+^ T cell response to the control recall antigens PPD and HA measured by IFN-γ release. These results were compared with healthy age-matched controls (n = 11) (Figure 3). CD4 and CD8 depletion experiments confirmed that these were CD4^+^ T cell responses (data not shown). There was no evidence that the patients were immunosuppressed; all subjects responded to at least one antigen. Cell proliferation was also assessed in parallel experiments as a measure of T cell activation, and again no evidence of immunosuppression was found pre-operatively (data not shown).

### Treg have a marked effect on anti-tumor immune responses in the blood of patients with CRC

**Figure 4:**
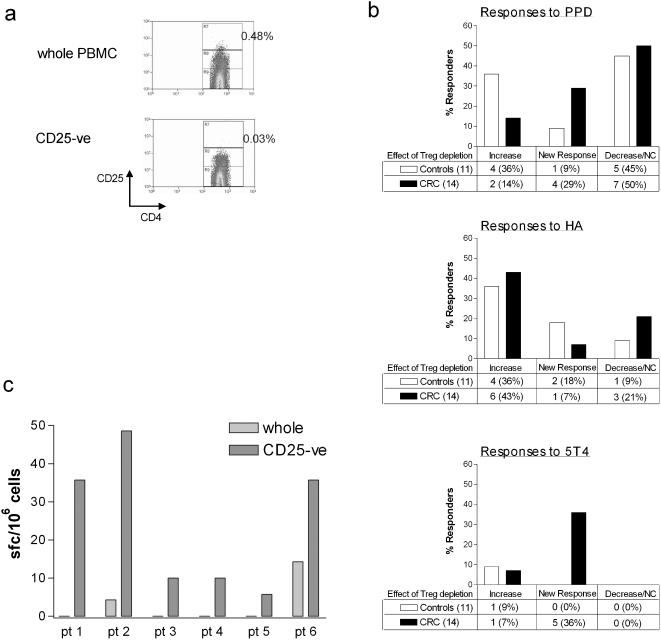
Depletion of Treg unmasks anti-tumor CD4^+^ T cell responses in CRC patients but not healthy controls. (A) PBMC were purified and two samples prepared: whole PBMC and PBMC depleted of CD25^hi^ cells. (B) Whole PBMC and CD25^hi^-depleted PBMC from 11 age-matched controls (open bars) and 14 CRC patients (solid bars) were set up in parallel in an IFN-γ ELISPOT assay (3.5×10^5^ cells/well) with PPD (top graph), HA (middle graph) or 5T4 (lower graph). The number of responders is shown for each antigen. In each case, the percentage on the left is the frequency of responders that increased after Treg depletion (Increase), the percentage in the middle is the frequency of non-responders that had a response after depletion (New Response) and on the right the frequency of responders that showed a decrease or no change in response after Treg depletion (Decrease/NC). The 95% Confidence Interval for paired differences of proportions between patients and controls is −0.83 to −0.22 revealing a significant difference for 5T4 responses. (C) The 6 CRC patients with responses to 5T4 are shown in more detail. The bars represent the spot-forming cells/10^6^ PBMC after subtraction of background spots, whole PBMC (light grey) and CD25^hi^-depleted PBMC (dark grey). Responses were observed only in CD25^hi^-depleted PBMC of patients 1–5, whilst a response was observed in both PBMC populations from patient 6. In all assays, wells were set up in duplicate.

A series of assays were performed on CRC patients (n = 14) and age-matched controls (n = 11) to determine whether removal of CD25^hi^ cells altered the immune response to recall antigens and the tumor-associated antigen 5T4. We optimized an isolation system designed specifically to deplete only the CD25^hi^ cells from our samples as outlined in methods (Figure 4a). Freshly-isolated PBMCs were used in *ex vivo* IFN-γ ELISPOT assays as outlined above to determine responses to the recall antigens and the tumor antigen 5T4, before and after depletion of Treg. On the whole, depletion of Treg uncovered some responses to recall antigens in CRC patients and controls (Figure 4b). However, in the case of the tumor associated antigen 5T4, there was a striking difference between controls and CRC patients. A 5T4-specific *ex vivo* response was found in 1/11 of the healthy controls and depletion of Treg enhanced this response. Whilst depletion of Treg augmented the 5T4-specific response in 1/14 of the CRC patients, depletion of this population led to the *unmasking* of a 5T4 response in 5/14 of the patients tested (95% Confidence Interval for paired differences is −0.83 to −0.22 revealing a significant difference in 5T4 responses between CRC patients and healthy controls). These data suggest that Treg are suppressing anti-5T4 immune responses specifically in patients with colorectal cancer. These six patients who demonstrated enhanced 5T4 responses had similar Treg frequencies in peripheral blood (range 0.82–2.35% mean 1.14%) to the frequencies measured in the entire CRC population (mean 1.13%). While IFN-γ-release, measured directly *ex vivo*, enabled anti-5T4 responses to be detected, we did not find vigorous proliferative responses to 5T4 in either CRC patients or controls. However, with several rounds of re-stimulation and the addition of IL-2, we have grown out HLA-DR-restricted 5T4-specific lines (data not shown).

## Discussion

We compared the frequency of carefully defined CD4^+^CD25^+^FOXP3^+^ regulatory T cells (Treg) in CRC patients with healthy age-matched controls and IBD patients. CRC patients have an increased frequency of Treg and there is evidence that these Treg target anti-tumor immune responses.

We assessed whether enhanced numbers of Treg in CRC patients correlated with general immunosuppression. A combination of IFN-γ ELISPOT assays and proliferation assays to monitor responses to the recall antigens PPD and HA, demonstrated equally robust responses in CRC patients and healthy controls, indicating that immune responses to non-tumor antigens are unimpaired in CRC patients.

The pattern of immune reactivity was strikingly different between CRC patients and healthy controls when immune responses to the tumor-associated onco-fetal antigen, 5T4, were compared. 5T4-specific responses were observed in a proportion of healthy controls, but these responses were largely unaltered by Treg depletion. In contrast, a 5T4-specific response was detected in more than a third of the CRC patients only after removal of Treg, suggesting that the Treg in CRC patients suppress the tumor-specific immune response. This group of CRC patients who demonstrated anti-5T4 responses after Treg depletion appeared to have similar Treg frequencies compared to the overall CRC group, although larger studies will be required to confirm this. Since 5T4-responses were not unmasked by Treg depletion in healthy controls, these data also indicate that Treg cells capable of suppressing 5T4-specific responses develop in response to the tumor. The precise mechanism through which this occurs is unclear at present, but it is possible that the cytokine milieu within the tumor promotes the generation of Treg recognizing tumor-associated antigens. Recent reports indicate a role for TGFβ in the expansion of Treg [24], [25]. In a rodent tumor model it has been shown that only dendritic cells (DCs) from tumor-bearing but not tumor-free animals promote proliferation of Treg. Interestingly DCs from tumor-free animals could expand Treg, but only if pre-incubated with tumor cell supernatants. These effects could be inhibited *in vitro* using anti-TGFβ antibodies [26]. Collectively, these data suggest that Treg require both a tumor antigen-specific stimulation and cytokines, such as TGFβ in order to proliferate and suppress anti-tumor immune responses.

5T4 is an attractive candidate for tumor vaccines as it is expressed in many epithelial tumors. However, this study suggests that Treg recognize the antigen and possibly play a role in suppressing anti-tumor responses, presumably to the detriment of the individual. Two other studies have demonstrated that Treg recognize tumor antigens (LAGE-1 and ARTC-1) in subjects with melanoma [27], [28]. It is important to ensure that vaccine strategies using tumor-associated antigens do not boost the regulatory arm of the immune system thereby inhibiting potential anti-tumor immunity. One approach may be to deplete Treg before vaccination, as recently demonstrated for renal cell carcinoma [29]. It will be of great interest therefore to determine in future studies whether such a strategy will result in measurable clinical responses in CRC patients.
